# Genetic Determinants of Time Perception Mediated by the Serotonergic System

**DOI:** 10.1371/journal.pone.0012650

**Published:** 2010-09-17

**Authors:** Olga V. Sysoeva, Alexander G. Tonevitsky, Jiří Wackermann

**Affiliations:** 1 Institute of Higher Nervous Activity and Neurophysiology, Russian Academy of Sciences, Moscow, Russia; 2 Russian Research Institute of Sport and Physical Education, Moscow, Russia; 3 Institute for Frontier Areas of Psychology and Mental Health, Freiburg im Breisgau, Germany; Duke University, United States of America

## Abstract

**Background:**

The present study investigates neurobiological underpinnings of individual differences in time perception.

**Methodology:**

Forty-four right-handed Russian Caucasian males (18–35 years old) participated in the experiment. The polymorphism of the genes related to the activity of serotonin (5-HT) and dopamine (DA)-systems (such as 5-HTT, 5HT2a, MAOA, DAT, DRD2, COMT) was determined upon the basis of DNA analysis according to a standard procedure. Time perception in the supra-second range (mean duration 4.8 s) was studied, using the duration discrimination task and parametric fitting of psychometric functions, resulting in individual determination of the point of subjective equality (PSE). Assuming the ‘dual klepsydra model’ of internal duration representation, the PSE values were transformed into equivalent values of the parameter 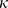 (kappa), which is a measure of the ‘loss rate’ of the duration representation. An association between time representation parameters (PSE and 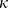, respectively) and 5-HT-related genes was found, but not with DA-related genes. Higher ‘loss rate’ (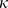) of the cumulative duration representation were found for the carriers of genotypes characterized by higher 5-HT transmission, i.e., 1) lower 5-HT reuptake, known for the 5-HTTLPR SS polymorphism compared with LL, 2) lower 5-HT degradation, described for the ‘low expression’ variant of MAOA VNTR gene compared with ‘high expression’ variant, and 3) higher 5-HT2a receptor density, proposed for the TT polymorphism of 5-HT2a T102C gene compared with CC.

**Conclusion:**

Convergent findings of the present study and previous psychopharmacological studies suggest an action path from 5-HT-activity-related genes, via activity of 5-HT in the brain, to time perception. An involvement of the DA-system in the encoding of durations in the supra-second range is questioned.

## Introduction

The internal representation of time, or ‘time perception’, is indispensable for orientation and purposeful action within one's physical and social environment. However, the physiological and psychological determinants of time perception, together with their underlying neural mechanisms, are still insufficiently understood. Further, there is substantial evidence for inter-individual differences in time perception, based upon both everyday experience and data from controlled experiments [1]–[3]. There is a large amount of literature hypothesizing the rôle of different neural transmission subsystems in time perception. Most reports connect mechanisms of time perception with the dopamine (DA) system [4]–[8]. However, there are also quite consistent findings that link duration representation with the serotonin (5-HT) system [9]–[12].

Several factors influence dopamine (DA) and serotonin (5-HT) transmission, particularly, the reuptake transporters, which remove the transmitter substances from the synaptic cleft, the number of receptors and their binding characteristics, as well as agents that regulate the catabolism of monoamines. Contemporary molecular genetic research has defined some genetic polymorphism associated with these factors: 5-HT and DA transport (5-HT-transporter-linked promoter region – 5HTTLPR [13], [14] and DAT VNTR [15]), receptor signaling (5-HT2a T102C [16] and DRD2 TaqI A [17]), and catabolism (monoamine oxidase A (MAOA) VNTR [18] and catechol-O-methyl transferase (COMT) V158M [19]).

The serotonin transporter protein 5-HTT is responsible for the reuptake of serotonin (5-HT) from the synaptic cleft, and determines the magnitude and duration of postsynaptic receptor-mediated signaling. The polymorphism in the promoter region of the 5-HTT gene has shown functional significance in coding high (L-allele) and low (S-allele) transporter production [13]–[14]. The variable number of tandem repeat (VNTR) polymorphism of the MAOA gene present in 2, 3, 3.5, 4, or 5 copies [18]: variants with 3.5 or 4 copies of the repeat sequence are transcribed more efficiently (‘high-expression variants’) than those with 3 or 5 copies of the repeat (‘low-expression variants’). The MAOA gene polymorphism is an X chromosome-linked polymorphism, meaning that only one copy is present in males. In the T102C polymorphism of the 5HT2a receptor gene, the base in nucleotide position 102 may be thymine (T) or cytosine (C), with three possible genotypes TT, TC or CC [16]. It has been shown [20] that the C allele has ca. 20% lower expression than the T allele. Therefore, higher 5-HT transmission is related to the T allele of 5-HT2a gene, the S allele of the 5-HTT gene and ‘low-expression variants’ of the MAOA gene.

The VNTR polymorphism of human DAT gene yields several alleles ranging from 3 to 11 repeats [15], alleles of 9 and 10 repeats being the most common [21]. Most studies reported an association of the 10-repeat allele with higher levels of expression of the gene [22]–[25], although some studies have reported higher levels of transcription associated with the 9-repeat allele [26] or no association between the VNTR and DAT density [27]. COMT degrades catecholamines such as dopamine. A polymorphism located at codon 158 of the COMT gene may contain the amino acids valine (*val*) or methionine (*met*). The *val* allele is 3 to 4 times as active as the *met* allele [28]. The presence of one or two A1 alleles in TaqI A polymorphism of the DRD2 gene was associated with reduced D2 receptor binding in all areas of the striatum, reaching statistical significance in the ventral caudate and putamen [17]. Therefore, higher DA transmission is related to A2 allele of DRD2 gene, 9 allele of DAT gene and *met* allele of COMT gene.

Contemporary methods of molecular genetics permit the investigation of the genetic basis of inter-individual differences in time perception by studying the association between gene polymorphism and duration representation characteristics. The aim of the reported study was to investigate the association of polymorphism of genes related to the activity of 5-HT- and DA-systems (such as 5-HTT, 5HT2a, MAOA, DAT, DRD2, COMT) with individual differences in duration representation. Based upon the review of literature, it is expected that both 5-HT and DA related polymorphisms would exhibit measurable differences in a time perception task. For that purpose we used a standard psychophysical duration discrimination paradigm, combined with data reduction and analysis relying upon the ‘dual klepsydra’ model (DKM) [29]. The DKM is based on a hypothesis of internal time representation in ‘inflow/outflow’ systems as, for example, externally driven and spontaneously de-exciting neural assemblies. This model permits to quantify the ‘loss rate’ of those ‘neural accumulators’ by a single parameter, 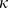 (kappa), which can be estimated both from duration discrimination [30] and duration reproduction data. The DKM parameter 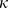 was shown to vary significantly with the phase of the circadian activation cycle [31]; to be sensitive to a neurochemical intervention affecting the 5-HT system [11]; and to differ significantly between two ethnically different populations, Swedish African compared to native Swedish population (reanalysis of data from [2] in [31]). All these findings indicate that 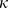 reflects state-dependent (externally induced or internally driven) changes and/or constitutional differences in time perception.

## Materials and Methods

### Volunteers

Forty-four right-handed Russian Caucasian males (mean age 22 years, SD 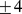 years) participated in the experiment, which consisted of a battery of cognitive tasks that were presented whilst EEG was recorded. The participants were selected from the database of the Russian Research Institute of Sport and Physical Education (Moscow), which contained subjects with DNA samples. The choice was made so as to balance the frequency of relatively rare genotypes with functionally different, but more frequent genotypes, to allow for their later comparison. To exclude possible effects of gender and handedness, only male, right-handed subjects were eligible for participation. The subjects were requested to have a good night sleep before the experiment. All experiments were conducted at about mid-day.

Only behavioural data from a duration discrimination task (described below, please see the Procedure section) are reported here. Thirteen subjects from the sample participated in a replication of the duration discrimination task, with the interval between sessions ranging from 2 weeks to 9 months.

The study was approved by the Russian Research Institute of Sport and Physical Education Ethical Committee. The aim and nature of the experiment was explained to the participants and all of them gave written informed consent before the experimental session.

### Genotyping

Genomic DNA was extracted from venous blood according to standard procedures. The 5-HTTLPR, MAOA VNTR, T102C 5HT2a, Val158Met COMT, TaqIA DRD2, DAT VNTR polymorphisms was evaluated as specified by previous published experimental protocols (specifically Refs. [32]–[37]).

### Procedure

**Figure 1:**
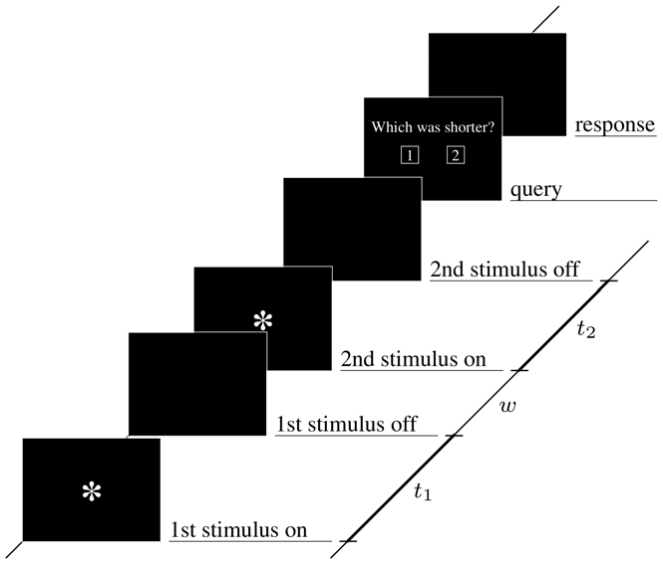
Duration discrimination paradigm. (Adapted from [37].)

A 2-alternative forced-choice duration discrimination task, similar as used in [30], was employed as part of the test battery in the experiment. The subjects had to compare two durations (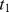 and 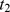), marked by the appearance of a visual stimulus on a computer monitor, observed from a distance of about 100 cm. The duration carrier stimulus was a gray asterisk (RGB = 160,160,160) of 15 mm diameter, displayed on a black background (Fig. 1). The inter-stimulus interval (ISI) was kept at a constant 0.9 s throughout. The subjects had to indicate which of the durations was shorter, choosing one of the displayed response boxes with a pointing device; a neutral response (‘apparently equal’) was not allowed. The interval from the end of the second interval to the query was 1.0 s. There was no fixed inter-trial interval; the subjects had to press a button on the response device to proceed to the next trial. No feedback was provided to the subjects during the session. Prior to the experiment, the subjects were verbally discouraged from sub-vocal mental counting or similar time-keeping strategies, such as hand or foot tapping.

The difference between the two durations, 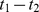, was varied at nine levels with a step of 0.4 sec, while the sum of the two durations was kept constant, 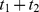 = 9.6 s. This design resulted in nine combinations of durations (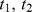), namely, (3.2, 6.4), (3.6, 6.0), (4.0, 5.6), (4.4, 5.2), (4.8, 4.8), (5.2, 4.4), (5.6, 4.0), (6.0, 3.6), and (6.4, 3.2 s). These combinations covered symmetrically duration ratios range from 1/2 to 2/1. For each of the nine combinations (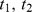) there were eight repetitions, presented in a randomized order. The entire block, comprising 9
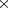
8 = 72 trials, took about 15–20 minutes.

### Analysis

In principle, the data analysis followed the same approach as that in described in [30]. Relative frequencies of the response ‘1’ (indicating that the subject perceived the 1st interval as shorter), were calculated separately for each subject and each of the durations pairs (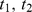). Gaussian psychometric functions were fitted to the data:
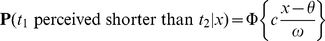
(1)where 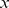 is a relative difference of the two durations,
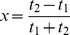
(2)In eq. (1) 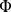 denotes the Gaussian cumulative distribution function, 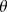 identifies the point of subjective equality (PSE), 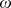 is a measure of discrimination uncertainty, and the constant 
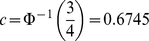
 (i.e., 75% quantil of the normal distribution) is introduced merely for convenience, to have 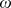 scaled to ‘just noticeable differences’ as units. As seen from eq. (1), the probability of the 1st and of the 2nd interval being perceived shorter is equal for 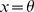: then 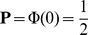. For the PSEs (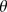 values) given by a psychometric function fit, pairs of subjectively indifferent durations 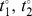 were calculated:
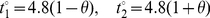
(3)The case 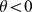 (observed in most subjects; see below, Results), i.e., 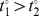, indicates a ‘subjective shortening in memory’ [38] of retained durations, a phenomenon which is naturally accounted for by the dual klepsydra model (DKM) of internal representation of temporal durations [30]. The corresponding value of the parameter 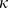 can then be estimated from the subjectively indifferent durations 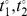 (details given in [30], p. 248). The case 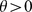 cannot be represented by the DKM, since 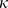 cannot attain negative values; for these cases we put, formally, 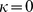. For descriptive purposes, the ratios of subjective equality (RSE)
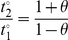
(4)were also calculated.

The data were compared between the subjects with different genotypes, using one-way ANOVA with subsequent post-hoc Fisher's Least Significant Difference (LSD) analysis, and 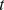-test for independent variables. For those 13 subjects who were available for the replication experiment, Pearson correlations between the measures obtained from the 1st and 2nd experiment were calculated, to assess the test–retest stability for the PSEs and 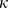
's.

**Figure 2:**
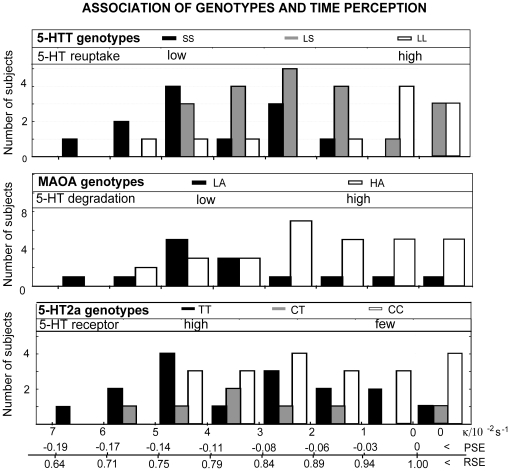
Association of genotypes and time perception. The distribution of 5-HT-related genotype carriers (vertical boxes of different shadings, number of subjects) in relation to the duration discrimination parameters, namely 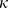, PSE and RSE (lower horizontal line) is shown. The predominance of SS genotype of 5-HTT gene, LA genotype of MAOA gene and TT genotype of 5-HT2a gene, i.e., genotypes related to higher 5-HT transmission, is clearly seen for the subjects with more negative PSE, and corresponding 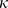 and RSE values.

Multiple regression analysis was done to examine the relationship between each dependent variable (PSE, RSE, or 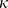) and set of independent variables, such as genotypes, related to 5-HT or DA systems. For each polymorphism type, the coefficients, indicating relative differences between genotypes, were assigned in accordance with the precedent within the literature. The *val* allele of COMT gene was claimed [28] to be 3 to 4 times as active as the *met* allele, therefore the weights 1, 2, 3 were assigned to genotypes *met*/*met*, *val*/*met*, *val*/*val*, respectively. Lesch [14] reported that S allele of 5HTT gene is about 20 times less productive compared to L-allele, therefore, 2, 3, 4 weights were assigned to SS, LS and LL genotypes, respectively. Polesskaya and colleagues [20] showed that the C allele of 5HT2a gene has 20% lower expression than the T allele, therefore the ratio between T and CC variants is about 5/4. According to [18, Fig. 2] the alleles with 4 copies of the repeat sequence are transcribed about 3 to 5 times more efficiently than with 3 copies, therefore, the weights 4 and 1 were assigned 4- and 3-copies alleles, respectively. Based on the study of Thompson and others [17] the approximate average ratio between density of DRD2 receptors for carriers of A1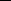 and A1+ variants of DRD2 gene was 3/2. According to study of Heinz and colleagues [23] individuals with the 9-repeat/10-repeat genotype had a mean 22% reduction of DAT protein availability in putamen compared with 10-repeat homozygous individuals. This ratio was approximated to be 4/5.

## Results

Molecular genetic analysis allowed to define the polymorphisms of 5-HTT, COMT, 5HT2a, DAT genes for all 44 subjects, DRD2 gene polymorphisms for 43 subjects, MAOA gene polymorphisms for 41 subjects. For reliable statistical comparison some rare genotypes with similar functional activity were merged together: TT and TC genotypes of 5HT2a were merged to T+, 10/9 and 9/9 variants of DAT gene were merged to 9+, A1A1 and A1A2 variants of DRD2 gene to A1+. Post-hoc data inspection revealed an atypical form of psychometric function in one subject, who seemingly misunderstood the instruction; this subject was excluded from further analysis. The numbers of subjects with particular genotypes are presented in Tables 1 and 2.

**Table 1:** The distribution of carriers of different genotypes of COMT, DAT and DRD2 genes in the sample.

COMT	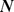	DAT	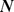	DRD2	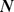
*val/val*	13	10/10 (9 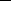 )	22	A1A1 (A1+)	4
*val/met*	20	10/9 (9+)	21	A1A2 (A1+)	15
*met/met*	11	9/9 (9+)	1	A2A2 (A1 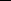 )	24

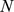
 = total number of subjects.

**Table 2:** 5-HTT, MAOA and 5-HT2a genotypes and duration representation parameters: PSE, RSE and 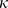.

	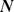	PSE			RSE			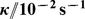		
		mean 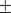 SE	min	max	mean 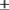 SE	min	max	median	min	max
5-HTT genotypes										
LL	11	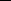 0.037 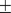 0.020	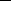 0.143	0.066	0.94 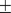 0.04	0.75	1.14	1.6	0.0	5.1
LS	20	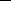 0.054 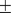 0.014	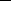 0.129	0.107	0.90 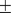 0.03	0.77	1.24	2.4	0.0	4.6
SS	12	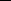 0.112 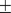 0.012	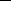 0.171	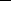 0.042	0.80 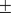 0.02	0.71	0.92	4.3	1.5	6.1
MAOA genotypes										
4-HA	27	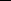 0.047 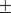 0.013	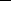 0.159	0.107	0.92 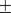 0.02	0.73	1.24	1.4	0.0	5.7
3-LA	13	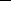 0.101 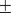 0.013	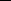 0.171	0.000	0.82 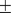 0.02	0.71	1.00	4.0	0.0	6.1
5-HT2a genotypes										
CC	20	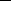 0.046 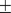 0.015	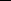 0.139	0.107	0.92 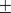 0.03	0.75	1.24	2.0	0.0	4.9
TC(T+)	7	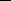 0.080 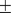 0.022	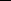 0.143	0.032	0.86 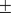 0.04	0.75	1.07	3.5	0.0	5.1
TT(T+)	16	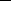 0.085 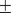 0.014	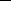 0.171	0.000	0.85 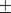 0.02	0.71	1.00	2.9	0.0	6.1
T+	23	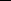 0.084 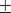 0.012	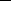 0.171	0.032	0.85 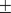 0.02	0.71	1.07	3.5	0.0	6.1

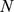
 = total number of subjects.

Table 2 contains descriptive statistic values for duration representation parameters (PSE, RSE and 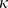) calculated for 5-HTT, MAOA and 5-HT2a genotypes.

One-way ANOVA with LL, LS and SS variants of 5-HTT gene as 3 levels of independent categorical factor 5-HTT revealed main effect of these gene on PSEs (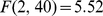, 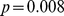), RSE (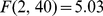, 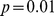) and 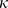 (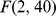 = 6.67, 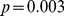) values. Post-hoc Fisher LSD tests revealed that PSE, 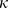 and RSE values were significantly different between SS genotype and two others (LL and LS). A 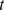-test for independent variables revealed a significant difference between the carriers of 3 and 4 variant of MAOA gene: the carriers of ‘low-expression variants’ of MAOA gene had smaller PSE (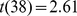, 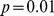), RSE (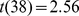, 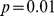) and, accordingly, greater 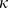 (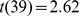, 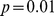) values than the carriers of ‘high-expression variants’. There were also significant differences between homozygote variants of 5-HT2a gene: T-carriers had more negative PSE (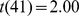, 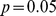), smaller RSE (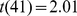, 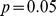), than CC-carriers.

Fig. 2 graphically presents the number of subjects of described 5-HT-related genotype (vertical boxes of different shadings) in relation to the duration discrimination parameters, namely 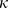, PSE and RSE (lower horizontal line). The predominance of SS genotype of 5-HTT gene, LA genotype of MAOA gene and TT genotype of 5-HT2a gene is clearly seen for the subjects with more negative PSE, and corresponding 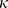 and RSE values.

No significant differences between time representation parameters were observed for DA-related genes.

Multiple regression analysis revealed a significant relationship between 5-HT-related genes and duration representation parameters (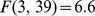, 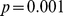 for PSE, 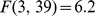, 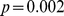 for RSE, 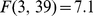, 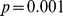 for 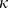). The multiple regression coefficients are significant for 5-HTT and MAOA (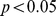) and marginally significant for 5-HT2a (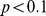). The multiple regression equations are
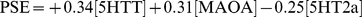


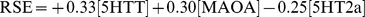


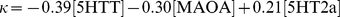
Multiple regression analysis did not reveal any significant relationship between duration representation parameters and genotypes related to dopaminergic system (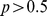).

Significant correlations between the first and second session (test–retest) were found: for PSE 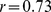, 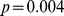, for kappa 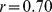, 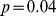.

## Discussion

The results of our study suggest that there is an association between 5-HT-related genes and internal (neural) representation of temporal durations, assessed by parameters derived from psychometric functions, and their DKM-based equivalent (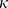). Precautions were taken against possible effects of confounding variables. Only male participants were recruited for the study, since previous studies demonstrated gender-related difference in the 5-HT-system functioning in human and animals (cf. [39]). To avoid possible effects of sleep deprivation [40], the subjects were requested to have a good night sleep. The experiments were conducted at the same time of day to eliminate or minimize a possible influence of circadian phase [31]. Data reduction and analysis relied upon a model that already has been successfully used in other studies [11], [30]. High test–retest correlations obtained in our study confirmed that the time representation parameters used for the given purpose, i.e., PSE and 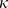, are stable characteristics of the subjects, and therefore provide a reliable basis for comparisons between carriers of different genotypes.

We found that genotypes that were characterised by a higher 5-HT transmission demonstrated a higher ‘loss rate’ of internal duration representation: 5-HTTLPR SS versus LL polymorphism, ‘low expression’ variant (3) versus ‘high expression’ variant (4) of MAOA VNTR gene, and T versus CC polymorphism of 5-HT2a T102C gene. In contrast, no association between time representation parameters and the dopamine (DA) related genes, such as DAT VNTR, DRD2 Taq1, COMT Val158Met polymorphisms, was observed in our study. It is known that MAOA also influences DA degradation, but to a lesser degree than 5-HT. Considering that no purely DA-related genes were shown to be associated with duration representation parameters, our study suggests that the duration representation in the supra-second domain may be not related to the DA-activity.

It is generally agreed that time perception exhibits a multi-regional structure, i.e. different mechanisms are responsible for subjective duration encoding in different time ranges [29], [41]. Most reports connect mechanisms of time perception with the dopamine DA system [4]–[8], [42]. Many pharmacological studies showed that substances influencing DA activity (such as haloperidol, which is a blocker of the DRD2 receptors) impair the processing of short durations (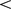
500 ms) [5]–[7], [43], [44], whereas the representation of longer durations is more dependent upon other cognitive processes, such as attention and memory, and therefore influenced by many other substances. There are also quite consistent findings that the representation of intervals 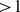 s is associated with serotonergic (5-HT) activity [9]–[12]. For example, Mitrani et al. [10] showed that LSD25 and mescaline specifically destroyed the time orientation, keeping the short duration (300–1000 ms) perception intact. These substances mostly activate the 5-HT system [45]. Other studies [11], [12] found an effect of psilocybin, a 5-HT receptor agonist, on time representation. In sum, pharmacological data indicate the relation of time representation and subjective time experience to DA- and 5-HT-related systems, the latter being mostly associated with representation of supra-second intervals.

A recent study [46], carried out on a selected population of women (synchronous swimmers), also revealed associations between genetic polymorphisms and individual differences in subjective time perception, suggesting that short duration perception (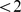 seconds) is related to the dopaminergic system, whereas the perception of longer durations is related to the serotonergic system. The study showed that genes related to the activity of the DA-system (COMT gene) was associated with the reproduction of 1–2 seconds intervals, whereas genes related to the activity of 5-HT system were associated with results in a time production task (1 minute interval), a subjective time flow questionnaire, and a current time orientation test. It is noteworthy that the association of the 5-HT-related genes with duration representation in the several seconds range was observed both for women [46] and for men (the present study). Therefore, we suggest that the pattern of results reported here—i.e., higher ‘loss rate’ of duration accumulation for carriers of genetic variants with higher 5-HT activity—also holds for women population. However, more studies are needed to investigate this issue further, keeping in mind that gender effects on time perception have been reported [47].

The major result of our study is the association of 5-HT related genes with time perception parameters, such as PSE, RSE, 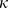. What does it mean in terms of neurobiology? As argued in [48], excitable cell ensembles can serve as putative integrators in the DKM scheme. We may hypothesise that ad hoc allocated local neuronal assemblies play the rôle of the ‘accumulators’, whereas the equilibrium state maintained by intra-assembly self-excitation corresponds to the ‘empty’ accumulator, and a temporary input from an extraneous source corresponds to the ‘inflow’ increasing the overall excitatory state of the assembly. The balance between self-excitation and de-excitation within the assembly ascertains its stability and determines its reactivity to the extraneous input. Specifically, the de-excitation rate, acting towards the functional equilibrium of the assembly, corresponds to the ‘loss rate’ in the DKM, represented by the parameter 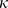. Our current study connects the parameter 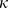 with the 5-HT transmission: the more 5-HT available for receptor activation (here 5-HT2a), the higher is 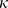. Therefore, the serotonergic system, known for its modulation effects on neuronal processes, can be proposed as important modulator of the de-excitation rate of the hypothetical ‘accumulators’. In contrast to passive physical models of ‘lossy integration’ (e.g. a charged capacitor–resistor circuit [29]), in a realistic neural implementation the ‘loss’ is an active process, acting as a clearance of previously allocated neural assemblies for re-use in newly arising time representation requests. The necessity of a clearance process follows naturally from the fact of limited resources of the central nervous system.

Our study may provide a new view on the psychobiological background of some individual differences. Many studies have reported an association between duration representation parameters and personality [49]–[51], specifically impulsivity [49], [52] and psychoticism [50]. Time perception was found to be altered in depressive states [53]–[56]: specifically, Gil and colleagues [56] reported more negative PSEs in a temporal bisection task for depressive patients, compared to healthy controls. Association of 5-HTT gene with neuroticism (negative emotionality), anxiety, hostility and depression was also shown [14], [39], [57], [58]: SS-carriers are more prone to negative emotions, depression and suicidality compared, to LL carriers. Therefore, it might be hypothesized that carriers of the low active genotype (SS) of 5-HTT gene have more negative PSE: this is exactly what is shown in our study. In our view, duration representation characteristics may thus provide the possible intermediate step between genetic variations and depression; this suggested link has to be substantiated by further research.

Our study might contribute to the understanding the brain systems involved in duration representation. A Positron Emission Tomography study revealed that 5-HTT availability was significantly reduced in the anterior cingulate cortex of individuals with impulsive aggression, compared to healthy subjects [59]. A recent study [60] reported that S-allele carriers had reduced grey matter volume in perigenual cingulate and amygdala. The involvement of cingulate cortex in duration representation is also discussed in the literature [61]–[64]. Therefore, this region may be part of the brain system through which the genetic base-to-duration representation association is realized.

In summary, we report the association between 5-HT-related genes and duration representation: carriers of genotypes related to the increased level of 5-HT transmission are characterized by higher loss rate (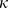) of internal duration representation, resulting in more pronounced ‘subjective shortening’ of elapsed time intervals. This result is in line with a recent finding [11] that psilocybin, an agonist of 5-HT receptors (therefore enhancing the 5-HT transmission), also temporarily increases 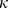 values. These convergent findings allow to suggest an action path from 5-HT-activity-related genes, via activity of 5-HT in the brain, to time perception. On the other hand, the absence of an association between DA-related genes and duration representation parameters questions the rôle of dopaminergic system in time perception in the supra-second range.
